# Interleukin-35 Is Involved in Angiogenesis/Bone Remodeling Coupling Through T Helper 17/Interleukin-17 Axis

**DOI:** 10.3389/fendo.2021.642676

**Published:** 2021-04-16

**Authors:** Hui Zhang, Yuxuan Li, Lin Yuan, Lutian Yao, Jie Yang, Liping Xia, Hui Shen, Jing Lu

**Affiliations:** ^1^ Department of Rheumatology and Immunology, The First Affiliated Hospital of China Medical University, Shenyang, China; ^2^ Department of Rheumatology and Immunology, Shengjing Hospital of China Medical University, Shenyang, China; ^3^ Department of Sports Medicine and Joint Surgery/Orthopedic, The First Affiliated Hospital of China Medical University, Shenyang, China

**Keywords:** interleukin-35, osteoclasts, bone resorption, angiogenesis, osteoporosis

## Abstract

**Objective:**

Osteoporosis is a common metabolic bone disease mainly involving bone remodeling and blood vessels. The current study aimed to explore the suppressive role of interleukin (IL)-35 in nuclear factor kappa-B ligand receptor activator (RANKL) and macrophage colony stimulating factor (M-CSF)-induced osteoclastogenesis and angiogenesis in osteoclasts.

**Methods:**

Osteoclasts differentiation were induced by incubation of mouse leukemic monocyte/macrophage cell line RAW264.7 cells in the presence of RANKL and M-CSF and was assessed with tartrate-resistant acid phosphatase (TRAP) staining assay. The viability and apoptosis of RAW264.7 was measured using CCK-8 assay and flow cytometry, respectively. The expression of angiogenic genes and proteins were measured using RT-PCR, Western blots and ELISA. The inhibition of Th17/IL-17 axis was examined using plumbagin, which was demonstrated as an IL-17A related signaling pathway inhibitor.

**Results:**

IL-35 inhibited the viability of RAW264.7 cells and promoted the apoptosis of RAW264.7 cells in a dose-dependent manner. Furthermore, IL-35 dose-dependently suppressed the expression of angiogenic markers including VEGF and its receptor. The suppressive effect of IL-35 was confirmed through the activation of Th17/IL-17 axis.

**Conclusions:**

We demonstrated for the first time the immuno-suppressive function of IL-35 on RANKL and M-CSF-induced osteoclastogenesis and angiogenesis through Th17/IL-17 axis. Therapeutic approach involving augmentation of IL-35 regulatory response may serve as a novel treatment option for osteoporosis, especially by suppressing bone resorption and angiogenesis.

## Introduction

Osteoporosis affects millions of people and is the most common bone metabolic disease worldwide. Bone is not only a skeletal system but also a vascularized tissue with a network of blood vessels and capillaries that provide nutrients and oxygen for bone development, which are regulated through the mediation of multiple signaling pathways between bone cells and endothelial cells (1, 2). Blood vessels play a crucial role in the development of osteoporosis and are formed through two different biological processes. In the early stage of embryogenesis, hemangioblasts are derived from mesodermal cells, which migrate to a specific site and aggregate to form the primary vessels in the process of vasculogenesis (3). Next, most of the new blood vessels sprout by the process of angiogenesis, which is coupled with an expansion of the existing angiogenic networks through a series of steps including proliferation, migration of endothelial cell, sprouting vessel pruning, and anastomosis (4, 5). Therefore, vasculature in bone is developed primarily by angiogenesis. Osteoclasts are the cells responsible for the breakdown of bone that is required for the bone turnover to maintain the skeleton healthy (6). In post-menopausal osteoporosis, osteoclasts are not kept in control by estrogen and so become overactive which contributes to the temporary imbalances of bone resorption and formation and leads to in overall net bone loss and osteoporosis (7). Previous study has demonstrated that vascular endothelial growth factor (VEGF) and its receptors fms-like tyrosine kinase (Flt-1) and fetal liver kinase (Flk-1) are expressed in osteoclasts (8). Aberrant expression of VEGF in bone can cause an increase in bone resorption by osteoclasts (9).

Interleukin (IL)-35 was first reported in 2007 that was composed of two components p35 and EBI3 (10). The same year, scholars identified that the IL-35, as an immunosuppressive cytokine, was a member of the IL-12 family of cytokines. And it could mediate the function of regular T cells (Tregs) and inhibit the proliferation of CD4^+^CD25^-^ effector T cells (Teffs) (11). IL-35 was first demonstrated that it was secreted by only FOXP3^+^ Tregs, but not effector T cells (Teffs), which was participated in multiple suppressive mechanisms (12). However, Shen et al. further demonstrated that IL-35 could also be produced by B cells and exerted the immunosuppressive function in autoimmune diseases (13). These findings revealed that IL-35 secretion might be influenced by immune microenvironments and a variety of stimulants. In recent years, IL-35 was found to be involved in in multiple inflammatory diseases, autoimmune diseases and cancers, including type I diabetes, hepatitis, non-small cell lung cancer (14–16). Previous study also reported that IL-35 participated in bone metabolism (17, 18). However, the possible mechanisms underlying this function have not been fully understood. In the current study, IL-35 could inhibit the viability of osteoclasts and suppressed angiogenesis by inhibiting the expression of VEGF and its receptor *via* Th17/IL-17 axis. These novel findings can help us to understand the mechanism between IL-35 and bone metabolism and may provide a new strategy for treating the skeletal diseases as well.

## Materials and Methods

### The Cells Culture

Mouse monocyte macrophage leukemia cell line RAW264.7 was used as osteoclasts precursor, which were purchased from the cell bank of Chinese Academy of Sciences. IL-35, RANKL and M-CSF were purchased from R&D system (USA). Fetal bovine serum (FBS) was purchased from Clark bioscience in the United States. DMEM high sugar medium was purchased from Hyclone (USA). Cell counting kit (CCK)-8 was purchased from Dojindo molecular technology (Japan). Plumbagin was purchased from Apexbio (USA). Acid phosphatase leukocyte (TRAP) kit was purchased from sigma (USA). TB Green™ Premix Ex Taq™ II (TliRNaseH Plus), Trizol reagent, PrimeScript™ RT reagent Kit with gDNA Eraser (Perfect Real Time) were purchased from Takara Biotechnology (Japan). Primers were synthesized by Zhenjiang aibimeng Biotechnology Co., Ltd. (China). VEGF enzyme-linked immunosorbent assay (ELISA) kit was purchased from Boster Biotechnology Co., Ltd. (China). Anti VEGF antibody and anti VEGF receptor 1 antibody were purchased from Abcam company in the United States. VEGF receptor 2 rabbit mAb was purchased from CST (USA). Annexin V-FITC cell apoptosis detection kit, BCA protein quantitative kit, SDS-PAGE gel preparation kit, and hypersensitive ECL luminescent kit were purchased from Beyotime Biotechnology (China).

### Osteoclasts Induction and Identification

The RAW264.7 cells line was cultured in a cell incubator with 37°C and 5%CO_2_. The morphology and growth were observed under the microscope every day. The cells were cultured in the presence of M-CSF (30 ng/ml) and RANKL (50 ng/ml) for 5 days, the culture medium was replaced every 2 days with fresh culture medium (DMEM high sugar medium, 10% FBS, 100 U/ml penicillin and 100 μg/ml streptomycin) supplemented with the agents described above. According to the above induction method, OCs were induced in the six-wells plate. After OCs were induced successfully, the cell climbing tablets were taken out and stained according to the instructions of TRAP kit for identification. The obtained mature OCs were stimulated with tumor necrosis factor (TNF)-α (20 ng/ml) for 2 h, and further cultured with IL-35 at various concentrations for 2 days.

### Cytotoxicity Assay to Measure the Viability of Osteoclasts

Cell viability was determined by CCK-8 assays. Briefly, mature osteoclasts were incubated in 96-well plate at a density of 5 × 10^3^/well, and then treated with different concentrations of IL-35 (0, 25, 50 and 100 ng/ml). Then the cells were treated with the CCK-8 reagents for 3-4 h. Ultimately, the optical density at 450 nm was measured by using enzyme labeling instrument (Thermo Fisher Scientific Spectrophotometer).

### Flow Cytometry to Detect the Apoptosis of Osteoclasts

TRAP-positive multinucleate cells were seeded into six-well plates (5×10^4^/well). Apoptosis of the cells was measured using Annexin V-FITC/PI Apoptosis Detection kit according to manufacturer’s protocol. Briefly, the cells were washed three times with cold PBS and centrifuged for 5 min at 1000 rpm/min. The cells were suspended in 100 μl of Binding buffer and were incubated with Annexin V-FITC (5 μl) and PI (10 μl) in the dark at room temperature for 10-20 min, then added 195 μl of Binding buffer, and the apoptotic cells were analyzed by a FACS Calibur flow cytometry. Annexin V-FITC single-positive cells are identified as early apoptotic cells. Annexin V-FITC and PI double-positive cells are identified as late apoptotic cells. Flow cytometry analysis: (1) Selected all particles, set forward scatted (FSC) and side scatted (SSC) to logarithmic axis mode for standby; (2) Set the horizontal axis and vertical axis to Annexin channel and PI channel respectively, and select double negative cell population; (3) In the double negative cell gate, set the horizontal axis and vertical axis to FCS and SSC respectively, change the data presentation mode to contour map, find the cell debris, draw the debris gate, and apply it to all cells; (4) In all cells, unselected the fragment gate to get all cells; (5) Set the horizontal axis and vertical axis as Annexin channel and PI channel, respectively, refer to the control group without IL-35 treatment, draw the cross gate to determine the ratio of different groups of cells.

### ELISA to Assess VEGF Levels in Culture Supernatants of Osteoclasts

The level of VEGF in osteoclasts supernatants was measured using ELISA kit. Osteoclasts were treated with stimulation of TNF-α (20ng/mL) for 2 h and then IL-35 at different concentrations (0, 25, 50 and 100 ng/ml) for 48 h, respectively. Osteoclasts supernatants were collected to analysis VEGF protein levels. The absorbance was measured at 450 nm using a microplate reader.

### RT-PCR to Assess the mRNA Expression of VEGF and Its Receptors

After incubation with stimulation of TNF-α for 2 h and IL-35 (0, 25, 50 and 100 ng/ml) for 48 h, total RNA was isolated from osteoclasts using Trizol reagent according to the manufacturer’s instruction. PCR reactions were conducted using a real time-PCR kit using following PCR conditions: 95°C 30 s 1 cycle; 95°C 5 s, 60°C 30 s 40 cycles; 95°C 5 s, 60°C 1 min, 95°C 1 cycle; 50°C 30 s 1 cycle. The primer sequences used for real time-PCR were as follows:

VEGF sense primer: 5^ʹ^-GCCAGAAAATCACTGTGAGCCTTGT-3^ʹ^,
anti-sense primer: 5^ʹ^-AGCTGCCTCGCCTTGCAACG-3^ʹ^;
Flt-1 sense primer: 5^ʹ^-CATGACGGAAGGAAGACAG-3^ʹ^,
anti-sense primer: 5^ʹ^-CAGGGGTAAGAGTATCAAATGG-3^ʹ^;
Flk-1 sense primer: 5^ʹ^-TGAGGAAAGGGTATTGGTG-3^ʹ^,
anti-sense primer: 5^ʹ^-AACAGTGGAGGCTATGTCG-3^ʹ^;
β-actin sense primer: 5^ʹ^-TGCGTGACATCAAAGAGAAG-3^ʹ^,
anti-sense primer: 5^ʹ^-AGAAGGAAGGCTGGAAAAG-3^ʹ^;

The relative expression of VEGF and its receptors including Flt-1 and Flk-1 were normalized with a β-actin internal control.

### Western Blot to Assess the Protein Expression of VEGF and Its Receptors

TRAP-positive multinucleate cells were stimulated with TNF-α (20 ng/ml, 2 h), and then treated with different concentrations of IL-35 for 48 h. Each group of cells was collected and were lysed by RIPA lysate, the protein was extracted. The BCA protein quantitative kit was used for protein quantification. The protein extract was isolated and transferred to a polyvinylidene fluoride (PVDF) membrane. Incubate the primary antibody at 4°C overnight, and then incubate the secondary antibody at room temperature for 2 h. The PVDF membrane was washed, and 1 ml ECL was added to the membrane. The gray-scale value of VEGF with its receptors and β-actin bands were analyzed, and protein expression was identified as the ratio of the gray-scale value of the VEGF and its receptors protein bands to the gray-scale value of the β-actin bands and scan the protein band on the BioRad chemidoctm MP imaging system with ECL luminescent kit.

### Analysis of Angiogenesis After Plumbagin Blockade

The osteoclasts were pretreated with 1 µM plumbagin for 24 h, then treated with TNF-α (20 ng/ml) for 2 h and IL-35 (0, 25, 50, 100 ng/ml) for 48 h. As mentioned before, ELISA, RT-PCR and Western blot were used to detect the expression of angiogenic molecules.

### Statistical Analysis

Data were analyzed using SPSS 23.0 software. Figures were created using Graph pad prism 7.0 software. The data are all expressed as Mean ± SD. One-way ANOVAs were used to analyze comparisons among groups and P<0.05 was considered significant.

## Results

### Identification of Osteoclasts

**Figure 1:**
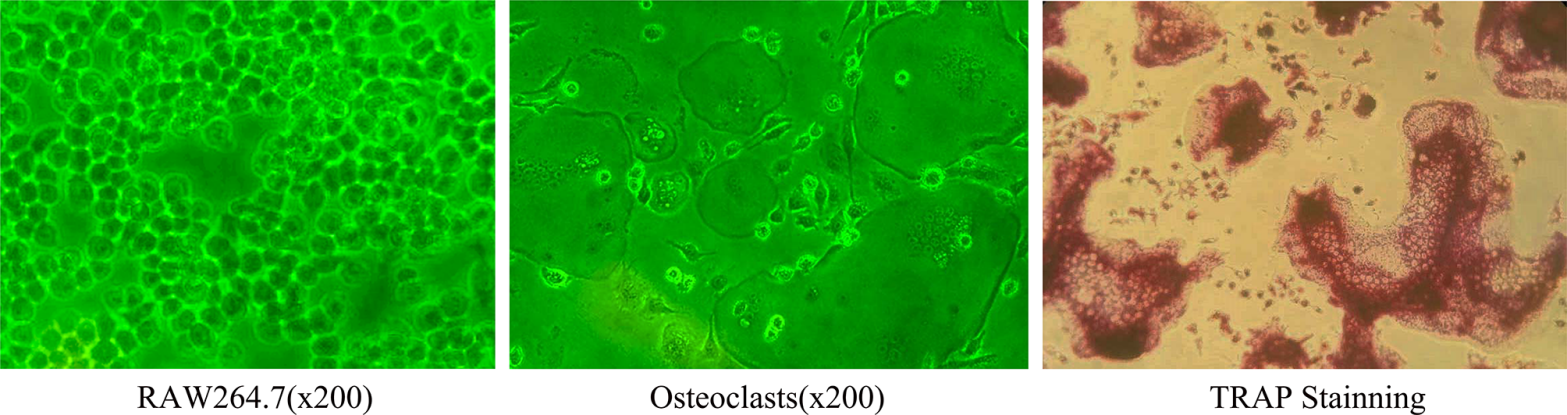
Under light microscope, RAW264.7 cells were round and mononuclear in shape and became osteoclasts after 5 days induction. At this time, the cells were large and multinuclear in shape, were surrounded by unfolding fold margin. TRAP staining was positive (Inverted phase contrast microscope x200).

The RANKL and M-CSF induced osteoclasts were stained with TRAP, and the TRAP-positive cells with more than 3 nuclei were considered as osteoclasts (**Figure 1**).

### IL-35 Significantly Inhibited the Viability of Osteoclasts

**Figure 2:**
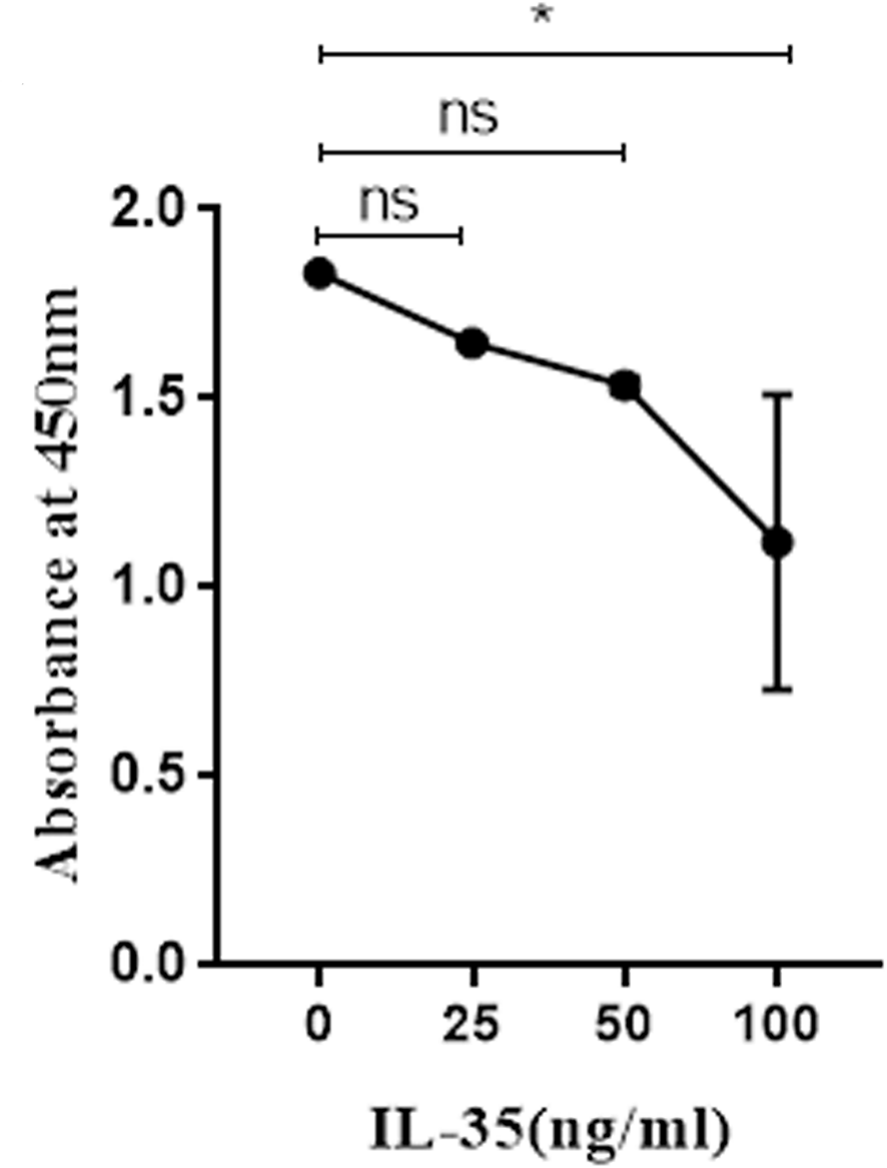
The effects of IL-35 at different concentration (0, 25, 50 and 100 ng/ml) on the viability of osteoclasts. Mean ± SD, n = 3. ^*^P < 0.05, ns, no significant.

To explore the effect of IL-35 on osteoclasts viability, we added different concentrations of IL-35 to osteoclasts which were successfully induced with RANKL and M-CSF. Compared with control group, IL-35 dose-dependently inhibited the viability of osteoclasts induced by RANKL and M-CSF. The results showed that IL-35 could decrease the viability of osteoclasts (**Figure 2**).

### IL-35 Significantly Promoted the Apoptosis of Osteoclasts

**Figure 3:**
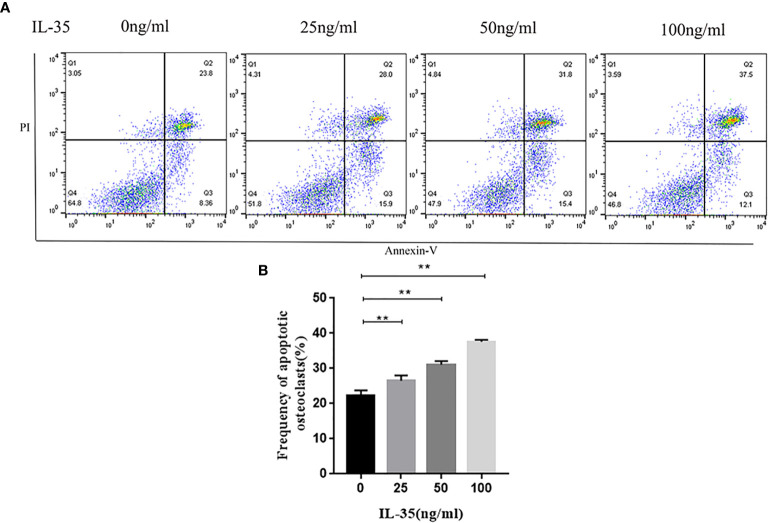
**(A)** Osteoclasts apoptosis in response to IL-35 at different concentration (0, 25, 50, 100 ng/ml) were determined by FCM. **(B)** Analysis of apoptotic rate. Mean ± SD, n = 3, ^**^P < 0.01.

Apoptosis is closely linked to angiogenesis. Therefore, we explored the effect of IL-35 on the apoptosis of osteoclasts. AnnexinV-FITC and PI doubled staining using FCM assay was used to explore whether IL-35 induced apoptosis in RANKL and M-CSF induced cell lines. The results showed that IL-35 could suppress the early and late apoptosis rate of osteoclasts compared to the control group in a dose-dependent manner (**Figure 3**).

### Detection of VEGF in the Supernatant of Osteoclasts by ELISA

**Figure 4:**
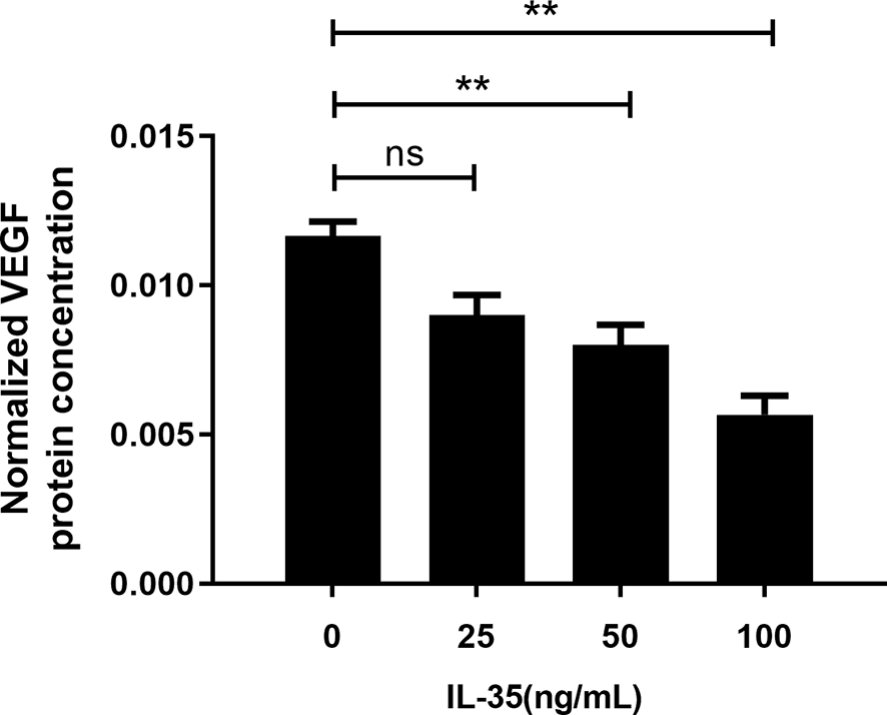
VEGF protein concentration after normalized with protein content in the supernatant of osteoclasts in response to IL-35 at different concentrations (0, 25, 50 and100 ng/ml). Mean ± SD, n = 3, ^**^P < 0.01, ns, no significant.

Furthermore, the quantitative measurement of VEGF protein levels in the osteoclasts supernatant was performed by ELISA assay. VEGF levels in the supernatant were normalized with the protein content of the cells considering IL-35 was toxic to the cells. After normalizing with the protein content of osteoclasts, the results showed that IL-35 at 50ng/ml and 100ng/ml significantly inhibited VEGF protein levels in osteoclasts supernatant after 48h incubation (**Figure 4**).

### IL-35 Inhibited the mRNA Expression of VEGF and Flt-1 in Osteoblasts

**Figure 5:**
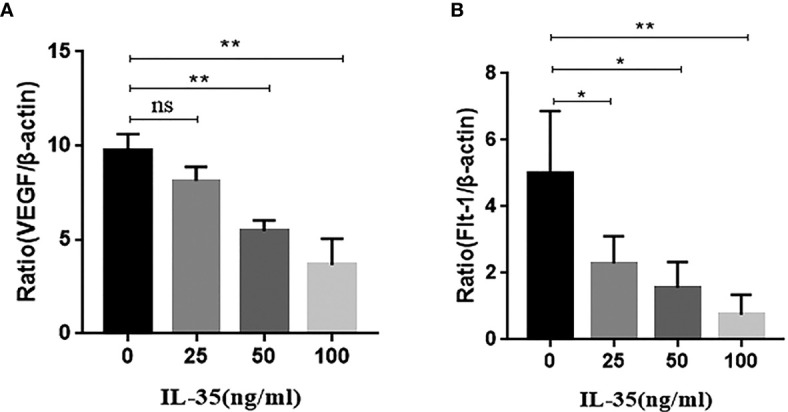
**(A)** VEGF mRNA expression was inhibited in response to IL-35 in a dose-dependent manner; **(B)** Flt-1 mRNA expression was inhibited in response to IL-35 in a dose-dependent manner. Mean ± SD, n = 3, *P < 0.05, **P < 0.01, ns, no significant.

We further investigated the effect of IL-35 on the mRNA expression of VEGF in osteoclasts. RT-PCR showed that IL-35 significantly suppressed the mRNA expression of VEGF and its receptor Flt-1 in cultured osteoclasts in a dose-dependent manner (**Figure 5**). However, mRNA expression of the other receptor of VEGF Flk-1 was not detected in each group (data not shown).

### IL-35 Inhibited the Protein Expression of VEGF and Flt-1 in Osteoclasts

**Figure 6:**
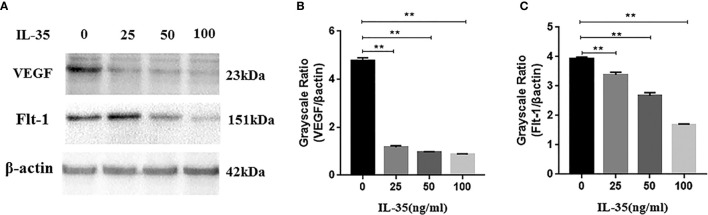
**(A, B)** VEGF protein expression is inhibited in response to IL-35 in a dose-dependent manner; **(C)** The receptor Flt-1 protein expression is inhibited in response to IL-35 in a dose-dependent manner. Mean±SD, n=3, **P < 0.01.

To confirm our findings, western blots was used to measure the VEGF and its receptors Flt-1, Flk-1 protein expression in the osteoclasts. Consistent with RT-PCR results, VEGF and Flt-1 protein expression in the IL-35-treated osteoclasts were lower than those in control group (IL-35 0 ng/ml). However, Flk-1 protein expression was still undetectable in each group. Together, these results strongly demonstrated that IL-35 could refrain angiogenesis in osteoclasts through inhibiting the expressions of vascular markers including VEGF and its receptor Flt-1 (**Figure 6**).

### Th17/IL-17 Axis Regulates VEGF Expression in Osteoclasts in Response to IL-35

**Figure 7:**
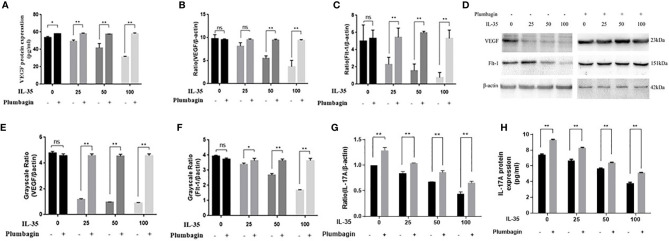
**(A)** VEGF protein expression in the supernatant of osteoclasts were determined by ELISA, without or with blockage of plumbagin. **(B, C)** RT-PCR analysis of VEGF and its receptor mRNA expressions in osteoclasts with or without blockage of plumbagin. **(D–F)** Western blot analysis of VEGF and its receptor protein expressions in osteoclasts with or without blockage of plumbagin. **(G)** IL-17A protein expression in the supernatant of osteoclasts were determined by ELISA, without or with blockage of plumbagin. **(H)** RT-PCR analysis of IL-17A mRNA expressions in osteoclasts with or without blockage of plumbagin. Mean ± SD. n = 6. ^*^P < 0.05, ^**^P < 0.01. ns, no significant.

To demonstrate the involvement of the pathway in the angiogenic effects of IL-35, we explored the effects of plumbagin which can inhibit the Th17/IL-17 related pathway. After blocking with plumbagin (1µM) for 24 h, VEGF and Flt-1 expression in osteoclasts were elevated in response to IL-35 at different concentrations (0, 25, 50 and 100 ng/ml). Furthermore, PCR and ELISA results showed that IL-17A secretion by osteoclasts in response to IL-35 was elevated after plumbagin blockage, which could be confirmed that Th17/IL-17 related pathway was blocked with plumbagin. The above results suggested that plumbagin blocked the anti-angiogenic effects of IL-35 in osteoclasts, demonstrating that IL-35 exerted its anti-angiogenic effects through Th17/IL-17 related pathway (**Figure 7**).

## Discussion

Osteoporosis is a common metabolic skeletal disease characterized by decreased bone microstructure and quantity, typically owing to enhanced osteoclastogenesis and/or increased osteoclastic bone resorption, leading to uncontrolled bone loss, which primarily occur in postmenopausal women. Bone homeostasis relies on the balance between the bone resorption by osteoclasts and bone formation by the osteoblasts (19). Osteoclasts are the bone cells which are responsible for resorption of bone and is a process important to maintaining the balance of healthy bones (20). Dysregulation of bone resorption by osteoclasts could contribute to osteoporosis and malignant osteolytic bone disease (21). Thus, osteoclasts have long been the crucial molecular targets for the development of therapeutic agents for the successful treatment of osteoporosis. Since bone is a rather vascularized system and angiogenesis is critical for osteogenesis, VEGF plays an important role in the development of the bone repair (22). VEGF is responsible for endochondral bone formation and required for effective coupling of angiogenesis and bone remolding during bone repair (23). It is known that VEGF is a crucial mediator of angiogenesis expressed by bone cells including osteoblasts, osteoclasts and chondrocytes and was shown to express VEGF receptor including Flt-1 and Flk-1 (9, 24, 25). Thus, the question we addressed concerned the possible mechanism of VEGF in RANKL and M-CSF induced osteoclasts.

IL-35 belongs to the IL-12 family, and its members include IL-12, IL-23, IL-27 and IL-35 (26). According to previous studies, IL-12 could inhibit the formation of bone marrow-derived osteoclasts in mice and promotes apoptosis of bone marrow cells (27, 28). Similarly, IL-27 can not only inhibit the production of human osteoclasts, but also play a stabilizing role in inhibiting bone erosion (29). In addition, IL-23 can also act as a biomarker of inflammation-induced bone disorders (30). However, it has not been fully understood whether IL-35 plays a role in skeletal system. In vivo study, IL-35 was confirmed to be positively correlated with bone mineral density, 25-(OH)VitD3 and be negatively correlated with β-isomerised carboxy-terminal cross-linking telopeptide of type I collagen, suggesting that IL-35 may be involved in the pathogenesis of skeletal system (17). Wanda Niedbala has confirmed that IL-35 can suppress the immune response by regulating the expansion of regular T cells and inhibiting the differentiation of Th17 cells (31). Furthermore, S. Wu reported that IL-35 could inhibit angiogenesis in fibroblast-like synovial cells through STAT1 signal transduction. Jiang et al. found that IL-35 inhibited angiogenesis and inflammation of rheumatoid arthritis by down-regulating basal and VEGF-induced angiopoietin-2 secretion and interfering with Ang2/Tyrosine kinase receptors signal transduction in human umbilical vein endothelial cells (32).

In our study, we demonstrated that IL-35 significantly inhibited the viability and promoted the apoptosis of RNAKL and M-CSF-induced osteoclasts. Our study clearly demonstrated that IL-35 strongly decreased the number of osteoclasts, suggesting IL-35 might inhibit the development of osteoporosis indirectly through bone mass. Furthermore, we were the first to report that IL-35 could inhibit the secretion of VEGF and suppress the expression of its receptor Flt-1. Flt-1 was expressed in monocytes and regulated their chemotaxis (33). Here, we also found that osteoclasts express Flt-1, but did not express the other receptor Flk, suggesting that, at the very least, Flt-1 was involved in not only angiogenesis but also osteoclastogenesis. Furthermore, previous studies demonstrated that Flk was expressed in mature osteoclasts, to some extent (9, 34). This might be the reason that we did not detect the Flk expression in our cells line. To explore the mechanism of the VEGF-VEGFR system in osteoclasts development and activity, we introduced plumbagin, a Th17/IL-17 axis specific inhibitor, in osteoclasts. Plumbagin is a natural bicyclic naphthoquinone derived from roots of the medicinal plant Plumbago zeylanica. The inhibitor has been known for its potent biological activities including anti-inflammatory, anti-tumor, and anti-bacterial activities (35). A recent study showed that it selectively inhibited IL-17 by CD4+T cells (36). In our study, IL-35 was found to inhibit the expression of VEGF and its receptor through Th17/IL-17 related pathway. The results showed that IL-35 could inhibit the angiogenesis in osteoclasts through Th17/IL-17 axis, proving that the Th17/IL-17 related pathway was activated to maintain osteoclasts commitment.

The traditional remedy of osteoporosis includes calcium plus active vitamin D, calcitonin, estrogen replacement therapy. However, in the previous extensive application, there are still some unavoidable concerns. First, most medicine can only attenuate symptoms, but cannot essentially solve the problem. Secondly, the treatment periods are all life long, and most patients have difficulties in economy and compliance. However, IL-35 therapy is designed to inhibit bone resorption through suppressing angiogenesis with fewer side effects. It can re-establish the stable skeletal system and bring promising sight for patients suffering from the osteoporosis for a long time. Under the advocation of “Precision Treatment” in recent years, IL-35 may have great prospects. In current study, there are still some limitations in related IL-35 immunotherapy. The *in vivo* potency of IL-35 immunotherapy needs to be further validated concerning its short half-life and instability. Furthermore, individual differences exist in the remedy, which needs demanding standardization of frequency and times of treatment.

## Conclusion

In conclusion, in current study we have confirmed for the first time that exogenous IL-35 can inhibit osteoclasts differentiation through decreasing the expression of VEGF with receptors *via* Th17/IL-17 axis, which suggesting that IL-35 is involved in angiogenesis/bone remodeling coupling through T helper 17/Interleukin-17 axis. However, the further improvement of this study requires more *in vivo* research.

## Data Availability Statement

The raw data supporting the conclusions of this article will be made available by the authors, without undue reservation.

## Author Contributions

Conception and design of study: JL and HZ. Acquisition of data: HZ, YL, and JY. Analysis and/or interpretation of data: HS, YL, LYao, and JY. Drafting the manuscript: HZ, YL, and LTY. Revising the manuscript critically for important intellectual content: JL, HS, and LX. Approval of the version of the manuscript to be published: HZ, YL, LYao, LTY, JY, LX, HS, and JL. All authors contributed to the article and approved the submitted version.

## Funding

This study was supported by a grant from the China National Natural Science Foundation (No.81471542), Postdoctoral Research Foundation of China (2019M661173, 2019M651171), and the Scientific Research Start-up Fund of Doctoral Researcher of Liaoning Province (20180540123).

## Conflict of Interest

The authors declare that the research was conducted in the absence of any commercial or financial relationships that could be construed as a potential conflict of interest.
